# Xeno-free cultured mesenchymal stromal cells release extracellular vesicles with a “therapeutic” miRNA cargo ameliorating cartilage inflammation *in vitro*

**DOI:** 10.7150/thno.77597

**Published:** 2023-03-05

**Authors:** Maria Elisabetta F. Palamà, Simona Coco, Georgina M. Shaw, Daniele Reverberi, Maddalena Ghelardoni, Paola Ostano, Giovanna Chiorino, Laura Sercia, Luana Persano, Maria Cristina Gagliani, Katia Cortese, Dario Pisignano, Josephine Mary Murphy, Chiara Gentili

**Affiliations:** 1 Department of Experimental Medicine, University of Genoa, Genoa, Italy; 2 Lung Cancer Unit, IRCCS Ospedale Policlinico San Martino, Genoa, Italy; 3 Regenerative Medicine Institute, National University of Ireland Galway, Galway, Ireland; 4 U.O. Molecular Pathology, IRCCS Ospedale Policlinico San Martino, Genoa, Italy; 5 Cancer Genomics Lab, Fondazione Edo ed Elvo Tempia, Biella, Italy; 6 Institute of Nanoscience (CNR-NANO), Pisa, Italy; 7 Department of Physics, University of Pisa, Pisa, Italy

**Keywords:** extracellular vesicles, osteoarthritis, mesenchymal stromal cells, microRNA, xeno free medium

## Abstract

**Rationale:** Mesenchymal stromal cells (MSCs)-derived extracellular vesicles (EVs) emerged as an innovative strategy for the treatment of chronic disorders such as osteoarthritis (OA). Biological activity of EVs is generally driven by their cargo, which might be influenced by microenvironment. Therefore, pre-conditioning strategies, including modifications in culture conditions or oxygen tension could directly impact on MSCs paracrine activity. In this study we selected an appropriate preconditioning system to induce cells to perform the most suitable therapeutic response by EV-encapsulated bioactive factors.

**Methods:** A xeno-free supplement (XFS) was used for isolation and expansion of MSCs and compared to conventional fetal bovine serum (FBS) culture. Bone Marrow-derived MSCs (BMSCs) were pre-conditioned under normoxia (20% O_2_) or under hypoxia (1% O_2_) and EVs production was evaluated. Anti-OA activity was evaluated by using an *in vitro* inflammatory model. miRNA content was also explored, to select putative miRNA that could be involved in a biological function.

**Results:** Modulation of IL-6, IL-8, COX-2 and PGE2 was evaluated on hACs simultaneously treated with IL-1α and BMSC-derived EVs. FBS-sEVs exerted a blunt inhibitory effect, while a strong anti-inflammatory outcome was achieved by XFS-sEVs. Interestingly, in both cases hypoxia pre-conditioning allowed to increase EVs effectiveness. Analysis of miRNA content showed the upregulation in XFS-hBMSC-derived EVs of miRNA known to have a chondroprotective role, such as let-7b-5p, miR-17, miR-145, miR-21-5p, miR-214-3p, miR-30b-5p, miR-30c-5p. Activated pathways and target genes were investigated *in silico* and upregulated miRNAs functionally validated in target cells. MiR-145 and miR-214 were found to protect chondrocytes from IL-1α-induced inflammation and to reduce production of pro-inflammatory cytokines.

**Conclusions:** XFS medium was found to be suitable for isolation and expansion of MSCs, secreting EVs with a therapeutic cargo. The application of cells cultured exclusively in XFS overcomes issues of safety associated with serum-containing media and makes ready-to-use clinical therapies more accessible.

## Introduction

Osteoarthritis (OA) is a complex and multi-factorial chronic joint disease, involving multiple tissues and processes. It compromises millions of people worldwide, representing a major health condition especially in elderly populations 1. Cartilage breakdown, ligament calcification and subchondral bone outgrowths are the pre-eminent pathological changes characterizing OA 2. Although the specific pathogenesis is uncertain, it is generally acknowledged that a link between OA progression and the production of pro-inflammatory cytokines exists 3. In this scenario, mesenchymal stromal cells (MSCs)-derived extracellular vesicles (EVs) represent a next generation strategy for OA treatment, thanks to their reparative and anti-inflammatory properties 3-5. MSCs have indeed been reported to mitigate OA through the secretion of both EV-encapsulated trophic factors, which support the endogenous cartilage repair, and anti-inflammatory cytokines 6. MSC-EVs support the maintenance of the tissue microenvironment, playing a crucial role when the tissue homeostasis is altered by a disease or a lesion 7. The biological effect of EVs is driven by their cargo, consisting of signalling molecules, such as proteins, lipids, DNA, RNA, miRNAs, surrounded by a phospholipidic bilayer. These miRNAs and proteins are involved in many cellular processes, such as communication, inflammation, metabolism, tissue repair and regeneration 8,9. Different compositions in miRNA cargo may significantly influence biological activities, being involved in epigenetic modification of cells 3. Since EV cargo mirrors the pathophysiology of the parental cells, the microenvironment may influence MSC paracrine signaling. Therefore, controlling MSC growth conditions by modifying the growth factor composition, oxygen tension, or local mechanical properties might directly shape MSC paracrine activity 10-12.

Hypoxia (*i*.*e.,* 1% O_2_) has been reported to enhance EV production in different cell types *in vitro* 13. It not only affects EV release, but also the cargo composition and, therefore, their therapeutic potential. In this framework, cell culture methods might be crucially important for EV production, especially for therapeutic purposes. Indeed media, often contains animal derivatives (*e.g.,* FBS), may be a source of contamination in the final product. Moreover, for large-scale production and clinical usage, generating completely animal-free products is essential.

In this study, a xeno-free supplement (XFS) was used for isolation and expansion of MSCs. As previously reported, XFS sustains cell proliferation and differentiation, besides improving EV release and their anti-inflammatory properties 14. However, the mechanism of action behind this biological effect still needs to be elucidated. Here, Bone Marrow-derived MSCs (BMSCs) were pre-conditioned under normoxia (*i.e.,* 20% O_2_) or under hypoxia and EV production was evaluated. Anti-osteoarthritic activity was also investigated by an *in vitro* inflammatory model 14. In addition, the ability of EVs to modulate pro-inflammatory cytokine production was explored on IL-1α-treated human articular chondrocytes. EV-miRNA cargo related to different pre-conditioning of secreting cells was also profiled and the most up-regulated ones were functionally validated in an osteoarthritic environment.

## Methods

### Cell culture

Human bone marrow stromal cells (hBMSCs) were derived from hip bone marrow aspirates of six healthy volunteers (average age 23 years, 4 males and 2 females), after informed consent and under ethical approval from Galway University Hospital and the National University of Ireland Galway Research Ethics Committees (Galway, Ireland). Cells were obtained and cultured as previously described 14 in Alpha-MEM-GlutaMAX (GiBCo, Waltham, MA, United States), supplemented with 100 U/mL penicillin-100 mg/mL streptomycin mixture (GiBCo) and (i) 10% FBS (Sigma-Aldrich, St Louis, MO, United States), 1 ng/mL Fibroblast Growth Factor-2 (FGF-2, Peprotech, London, United Kingdom) for standard culture conditions, (FBS-hBMSCs) or (ii) Xeno-free Purstem supplement (XFS-hBMSCs, patent No. PCT/EP2015/053223) 15.

Human articular chondrocytes (n = 10, average age 81 years, 5 males, 5 females) were obtained from femoral condyles of patients undergoing partial knee arthroplasty, as previously described 14. For each patient an informed consent was obtained, and the present study has been approved by the Ethical Committee of San Martino Hospital (CER Liguria: 372/2019 Genoa, Italy).

### hBMSCs pre-conditioning

hBMSCs were cultured until 60% confluence. Then, the medium was discarded and, after three PBS 1X washes, replaced with α-MEM-GlutaMax medium with only 100 U/mL of Penicillin-100 mg/mL Streptomycin (basal medium). Cells were maintained under normoxic (20% O_2_ and 5% CO_2_ at 37 °C) or hypoxic (1% O_2_ and 5% CO_2_ at 37 °C in a hypoxic incubator, Eppendorf, Hamburg, Germany) conditions, for 72 h. Conditioned media (CM) were collected and processed as described below.

### Propidium Iodide (PI) and FITC-Anti-annexin-V Apoptosis Detection

Viability of secreting hBMSCs was investigated by flow cytometry (BD FACSAria II, BD Biosciences), with double staining for Annexin-V and Propidium Iodide (PI). The Annexin-V apoptosis assay kit (BD Pharmingen, San Diego, CA, United States) was used, according to the manufacturer's instructions.

### Separation of Extracellular Vesicles from hBMSCs

EVs were separated from CM after high-speed differential centrifugations. CM was collected and immediately centrifuged at 300 g for 10 min at 4 °C to eliminate dead cells and debris, and at 2,000 g for 20 min at 4 °C to get rid of apoptotic bodies. Clarified CM was then ultracentrifuged at 10,000 g for 40 min at 4 °C, and finally at 100,000 g for 2 h at 4 °C, to pellet medium-size EVs and small-size EVs (sEVs), respectively. sEV pellets were washed with PBS 1X and finally resuspended in 0.22 μm-filtered PBS 1X to obtain normoxia or hypoxia derived FBS-hBMSC-sEVs (namely Normo FBS-sEVs and Hypo FBS-sEVs, respectively) and normoxia or hypoxia derived XFS-hBMSC-sEVs (namely Normo XFS-sEVs and Hypo XFS-sEVs, respectively). A Beckman Coulter ultracentrifuge (Beckman Coulter Optima XPN-100 ultracentrifuge; Beckman Coulter) was used with swinging bucket rotors type SW41Ti and SW55Ti.

The concentration of membrane-bound protein on the surface of freshly isolated, intact hBMSC-EVs was measured using BiCinchoninic Acid (BCA) assay (Thermo Scientific Pierce, Rockford, IL, USA), following manufacturer's instructions. Protein content was normalized on cell number (1x10^6^).

### Nanoparticle Tracking Analysis

EV samples were characterized and quantified by Zetaview (Particle Metrix GmbH, Germany) Nanoparticle Tracking Analysis (NTA), equipped with a sample cell and two lasers (488 nm and 640 nm). The software used was Zetaview 8.05.14_SP7. After calibration with 100 nm polystyrene beads, samples were diluted in 0.22 μm-filtered PBS 1X and injected using a 1 ml syringe. Size distribution analyses of 11 different positions were performed for each sample on at least three different sEVs preparations.

### Non-conventional flow cytometry

For flow cytometry characterization, sEVs were suspended in filtered PBS/EDTA, distributed in flow cytometry tubes (100 μl/tube) and stained with 1 μM CFDA-SE (VybrantTM CFDA SE Cell Tracer Kit, Thermo Fisher Scientific) at room temperature (RT) to visualize intact vesicles. Since CFDA-SE diffuses within vesicles only at RT, a control at 4 °C was also included. A mixture of fluorescent beads with a diameter from 100 to 900 nm (Megamix-Plus FSC and Megamix-Plus SSC, Biocytex, France) was used to discriminate sEV size. Expression of typical EV markers, such as CD9 (APC Mouse Anti-Human CD9, 312108, Biolegend, San Diego, CA, USA), CD63 (PE-CyTM7 Mouse Anti-Human CD63, 561982, BD Biosciences) and CD81 (BV421 Mouse Anti-Human CD81, 740079, BD Biosciences) was evaluated within the CFDA-SE positive events. All the evaluations were performed using a BD FACSAria II (BD Biosciences) and Flow Jo software for post-analyses.

### Labeling and Internalization of hBMSC-EVs by hACs

Extracellular vesicle uptake was monitored *in vitro* by staining FBS-sEVs and XFS-sEVs with PKH67 (Sigma-Aldrich), according to the manufacture's protocol. Staining was stopped by adding an equal volume of 1% bovine serum albumin (BSA) and the sEVs were ultracentrifuged to remove unbound dye. Chondrocytes (OA hACs), plated on cell culture slides were treated with 200 U/mL IL-1α (Peprotech, London, United Kingdom) overnight to mimic an inflammatory condition *in vitro*. After pre-treatment, 1 μg/mL of stained Normo or Hypo FBS- or XFS-sEVs was added to the cells for 3 h prior to fixation with 3.7% paraformaldehyde (PFA) and staining with 5 U/mL phalloidin-594 (Life-Tech) to visualize the cytoskeleton. Nuclei were stained with DAPI (Sigma-Aldrich) and cover slipped with an aqueous mounting. Slides were observed at different magnifications and images acquired with the Axiovert 200M microscope (Carl Zeiss, Oberkochen, Germany).

### Confocal microscopy

Laser scanning confocal microscopy was performed by a Leica SP5 inverted confocal microscope with a 40× (N.A. 1.24) oil immersion objective. Laser beams with 405 nm, 488 nm, and 561 nm excitation wavelengths were used for DAPI, PHK67-FITC, and Alexa Fluor 594 imaging, respectively. The fluorescence emission was collected in the range: 415-470 nm for DAPI, 500-555 nm for PHK67-FITC, and 570-670 nm for Alexa Fluor 594, by means of three photomultiplier tubes. The diameter of the detection pinhole was set to the size of 1 Airy. *z-*stacks of FBS-sEVs and XFS-sEVs were performed upon acquiring 70 slices of 0.13 µm, each slice being the average of four laser scans. Single confocal sections and *z*-stack data files were processed and analyzed by the ImageJ software.

In order to evaluate the nucleus-exosomes three-dimensional distance (*D_xyz_*), fluorescence *z*-stacks of each sample type were analyzed by considering a specific volume including a single cell with its vesicles. The three-dimensional distance between the nucleus centre and the exosome centre was measured as:



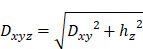



where *D_xy_* is the distance in the *x*-*y* plane and *h_z_* is the distance on the *x-z* or *y-z* plane. Moreover, the exosomes three-dimensional density was evaluated as the ratio between the number of exosomes and the cell volume (mm^3^) enclosing the nucleus and the vesicles. Five regions of interest were analyzed for each sample type.

### Electron Microscopy

FBS-sEV and XFS-sEV preparations were fixed by adding 2% PFA in 0.1 mol/L phosphate buffer (pH 7.4) in the same volume of EV resuspension buffer. Five μl drops of EVs were then adsorbed for 10 min on formwar-carbon-coated copper grids. Subsequently, grids were rinsed in PBS and negatively stained with 2% uranyl acetate for 5 min at room temperature. Stained grids were finally embedded in 2.5% methylcellulose for improving preservation and air-dried before examination. Electron micrographs were acquired using a Hitachi 7800 120 kV electron microscope (Hitachi, Tokyo, Japan) working at 100 kV, equipped with a Megaview G3 digital camera and Radius software (EMSIS).

### Western blot

Isolated sEVs were resuspended in RIPA buffer (1% NONIDET p-40, 0.1% SDS, 0.1% Sodium deoxycholate, protease inhibitor cocktail 1X, in PBS pH 7.5) and 2 μg protein for each sample were loaded on 4%-12% NuPAGE Bis-Tris gel (Life Technologies, Carlsbad, CA, USA). Electrophoresis was performed at 150 V and proteins were blotted on a polyvinylidene fluoride (PVDF) membrane (Millipore, Burlington, MA, USA). Blot membranes were incubated overnight at 4 °C with specific primary antibodies for: anti-human CD9 (1:1000 dilution, ab92726, Abcam, Cambridge, UK), anti-human CD63 (1:1000 dilution, 10628D, Invitrogen, Waltham, MA, USA), anti-human CD81 (1:5000 dilution, 555675, BD Biosciences), anti-human syntenin-1 (1:1000 dilution, ab133267, Abcam), anti-flotillin-1 (1:10.000, ab133497, Abcam). A specific HRP-conjugated secondary antibody (1:2000 dilution, Cell Signaling Technology, Danvers, MA, USA) was used for the detection. Positivity was highlighted by providing the substrates for the chemiluminescence reaction of HRP (Amersham ECL Prime Western Blotting Detection Reagent, GE Healthcare, Chicago, IL, USA) and impressing a photographic sheet by autoradiography (GE Healthcare). Images were scanned using the Epson perfection 1260 scanner. Gel running was performed under non-denatured conditions for the detection of CD9, CD81 and CD63 and under denatured conditions for other markers.

To investigate the effect of sEVs on OA-hACs, confluent hACs were treated for 16 h with 200 U/mL IL-1α and 1 μg/mL Normo or Hypo FBS- or XFS-sEVs. A negative control without IL-1α or sEVs and a positive control treated only with 200 U/mL IL-1α were also included. Conditioned media were collected, and cells were scraped in RIPA buffer to obtain cell lysates. Western blot was performed by incubating membranes with specific primary antibodies for IL-6 and IL-8 (1:200, Santa Cruz Biotechnology, Santa Cruz, CA, USA) and COX-2 (1:500, Abcam, Cambridge, UK). Images were scanned using the Epson perfection 1260 scanner and band densities quantified using Fiji-Image J software. All the analyses were performed on a minimum of four pools of three primary hACs each.

### RNA Extraction and Real-Time Quantitative Reverse Transcription Polymerase Chain Reaction (qRT-PCR)

Confluent hACs were treated for 16 h with 200 U/mL IL-1α and 1 μg/mL Normo or Hypo FBS- or XFS-sEVs. A negative control without IL-1α or sEVs and a positive control treated only with 200 U/mL IL-1α were also included. Cells monolayers were collected in TRIzol Reagent (Thermo Fisher Scientific, Waltham, MA, USA) and total RNA was extracted following manufacturer's instructions. Quality control of RNA concentration and purity were assessed using BioSpectrometer (Eppendorf, Hamburg, Germany). Complementary DNA (cDNA) was synthetized starting from 2 μg of total RNA using SuperScript™ IV VILO™ Master Mix (Thermo Fisher Scientific, Waltham, MA, USA) according to manufacturer's instruction. Transcript levels of *IL-6, IL-8, COX-2*, *COL1A1* (type I collagen), *COL2A1* (type II collagen) and *COL10A1* (type X collagen) genes were measured by real-time quantitative PCR (qRT-PCR) using BlasTaq™ 2X qPCR MasterMix (abmgood, Richmond, BC, Canada) on 7500 Fast Real-Time PCR System (Applied Biosystems, Foster City, CA, USA).

The house-keeping gene *GAPDH* was used as the endogenous control for normalization. The selected human-specific primer sequences are reported in Table 1. Data were analyzed with 2^-ΔΔCt^ method and reported as fold change/GADPH.

### NF-κB Nuclear Translocation

Four different pools of hACs were cultured in 24-well plates on glass coverslips (20,000 cells) and treated for 16 h with 200 U/mL IL-1α and 1 μg/mL Normo or Hypo FBS- or XFS-sEVs. Cells were fixed in 3.7% PFA for 10 min at RT and permeabilized with 0.5% Triton X-100 in PBS for 10 min at 4 °C. Non-specific binding was prevented by incubation with 20% goat serum (EuroClone) in PBS for 30 min at 4 °C. Slides were incubated with a rabbit anti-NF-κB p65 antibody (1:400; Cell Signaling, Danvers, MA, USA) overnight at 4 °C, followed by a goat anti-rabbit Alexa fluor-488 (Life Technologies, Grand Island, NY, United States) for 30 min at 4 °C. Coverslips were mounted with an aqueous mounting and images acquired using AxioPhot microscope (Carl Zeiss). A quantification was also performed counting cells with positive nuclei on at least five different areas for each experiment. Four independent experiments on pools of three hACs primary cultures were performed.

### PGE2 quantification

OA-hACs CM were assayed for PGE2 content using a PGE2-specific competitive EIA kit-Monoclonal (Cayman Chemical, Ann Arbor, MI, USA) according to the manufacturer's instructions. Each sample was measured in triplicate in two dilutions. Data were normalized to the IL-1α positive control.

### MiRNA profiling by microarray

For RNA extraction Normo and Hypo FBS- and XFS-EVs were suspended in 1 ml of Qiazol reagent (Qiagen, Limburg, The Netherlands). EV-RNA was extracted with the miRNeasy Micro Kit (Qiagen) according to the manufacturer's instructions and stored at -80 °C. MiRNA content was evaluated by the Qubit® 2.0 Fluorometer using the Qubit® microRNA Assay Kit (Thermo Fisher Scientific) and small RNA quality and size were assessed by Agilent 2100 Bioanalyzer using the Small RNA chip (Agilent Technologies, Santa Clara, CA, USA). The expression of 2,549 EV-miRNAs was investigated by microarray using SurePrint Human miRNA platform (Agilent Technologies; AMADID: 070156). Fifty ng of EV-RNA and Spike-in controls were dephosphorylated and labelled with Cyanine 3-pCp. Then, Cy3-miRNA samples were purified using Micro Bio-Spin P-6 Column (Bio-Rad Laboratories, Inc., Hercules, CA, USA) and hybridized with a synthetic DNA poly-A oligonucleotide 3' labelled pCp-Cy3 (50 amol, TIB Molbiol SRL, Genoa, Italy) on the microarray slide. Fluorescent signals were then acquired by a G2565CA scanner (Agilent Technologies) and data were extracted by Feature Extraction software v.9.5.3.1 (Agilent Technologies). Raw data were processed using the limma R package for microarray analysis. Background correction and between-array normalization were carried out using the *normexp* method, with an offset = 20, and the *quantile* method, respectively. A log(fold change)>0.378 was assumed to represent upregulated miRNAs, while miRNAs with log(fold change)<-0.378 were defined as downregulated. MiRNAs with p < 0.01 (-log10=2.0) were considered statistically significant. Raw and processed data are uploaded at the Gene Expression Omnibus repository (http://www.ncbi.nlm.nih.gov/geo/; GEO ID: GSE209585). The list of upregulated and downregulated miRNAs was loaded on DIANA miRPath 3.0 16, to perform Gene Ontology analysis on target genes. Pathway involvement was firstly investigated by using the algorithm for KEGG (Kyoto Encyclopedia of Genes and Genomes) pathway database examination. Target genes were predicted *in silico* by using miRsystem 17.

### Transfection of chondrocytes with double-stranded (ds) miRNA mimic

HACs were seeded in 24-well plates (2.5 x 10^5^ cells/well) and incubated for 24 h. Cells were then transfected with the following mirVana miRNA mimics: hsa-miR301a-3p, hsa-let7b-5p, hsa-miR145-5p, hsa-miR21-5p, hsa-miR30b-5p, hsa-miR30c-5p or a negative control (scramble) (100 nM, Thermofisher Scientific). Transfection was performed by using Lipofectamine 3000 (Invitrogen) in Optimem medium (GiBCo) and according to the protocol provided with the transfection reagent. Transfection efficiency was verified 48 h later, before performing further experiments. Cells were collected in QIAzol® reagent (Qiagen, Hilden, Germany) and RNA extracted by using miRNeasy Micro kit (Qiagen). TaqMan™ Advanced miRNA cDNA Synthesis Kit (Applied Biosystem) and TaqMan™ Advanced miRNA Assay (Applied Biosystem) were respectively used to perform reverse transcription and PCR following the manufacturer's instructions.

To perform the miRNA functional assay, after transfection for 48 h, medium was removed, and cells were stimulated with 200 U/mL IL-1α for 16 h and then total RNA prepared from chondrocytes was used to check the expressions of *IL-6, IL-8, COX-2, COL1A1, COL2A1, COL10A1, SOX9* (SRY-box transcription factor 9) and *RUNX2* (Runt-related transcription factor 2). Sequences of selected primers are listed in Table 1.

### Statistical analysis

Data were analyzed with GraphPad Prism 9.3 software (GraphPad Software, Inc., San Diego, CA, USA). One-way ANOVA test was performed for western blot on IL-6, IL-8, and COX-2, real time-PCR on *IL-6, IL-8, COX-2, COL1A1, COL2A1, COL10A1* and *SOX9*, NF-κB nuclear translocation, PGE2 ELISA quantification and miRNA functional tests. Two-way ANOVA was used for statistical analysis of cell viability, BCA protein assessment, NTA analysis, non-conventional flow cytometry on sEVs (MFI evaluation) and miRNA comparison. Level of significance was set at p < 0.05 (*p < 0.05, **p < 0.01, ***p < 0.001, ****p < 0.0001). The number of data used for the statistical analyses is reported in the figure legends and corresponds to independent experiments. Data are shown as mean ± SD.

## Results

### Hypoxic pre-conditioning of hBMSCs enhances sEV protein enrichment

**Figure 1:**
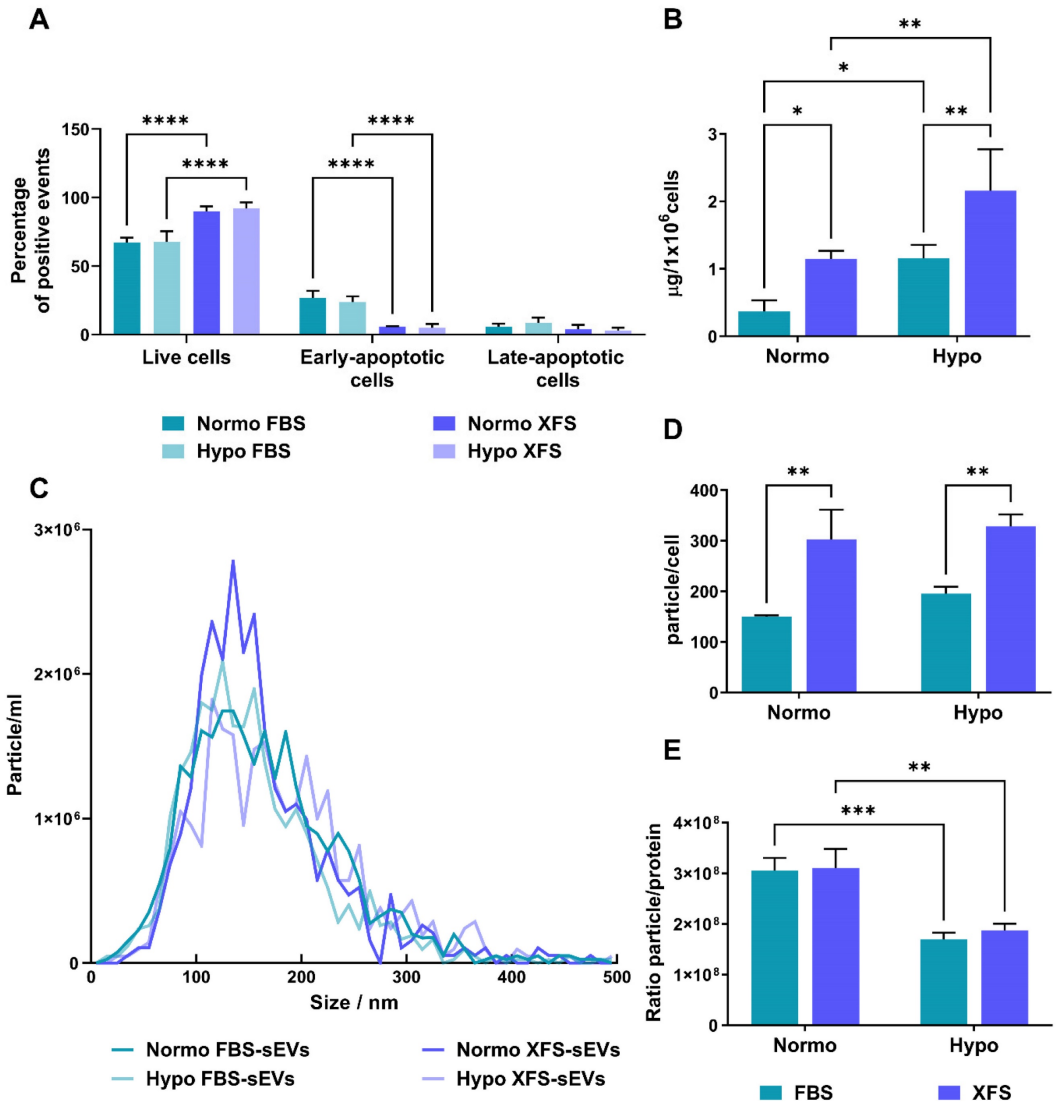
**Characterization of secreting cells and quantification of sEVs**. **(A)** Evaluation of apoptosis on secreting cells by flow cytometry on both FBS and XFS-hBMSCs after 72 hours pre-conditioning in both normoxic (20% O_2_) and hypoxic (1% O_2_) culture conditions. Concurrent staining with FITC-anti-annexin V and PI was used. Error bars represent S.D. (****) p < 0.0001, two-way ANOVA. **(B)** Quantification of protein content on Normo and Hypo FBS-sEVs and Normo and Hypo XFS-sEVs by BCA assay. Data are normalized on cell number (μg/10^6^ cells). Error bars represent S.D. (*) p < 0.05, (**) p < 0.01, two-way ANOVA. **(C)** Nanoparticle Tracking Analysis (NTA) measuring size distribution of FBS- (green) and XFS-sEVs (blue) under normoxia ad hypoxia. **(D)** Comparison of particle number expressed as particles/cell. Error bars represent S.D. (**) p < 0.01, two-way ANOVA. **(E)** Ratio particle/protein. Error bars represent S.D. (**) p < 0.01, two-way ANOVA. Data are representative of at least three independent experiments.

Changes in oxygen concentrations might affect stem and progenitor cells 18,19 and this can be mirrored in the secreted EVs. On this basis, we evaluated whether hypoxic conditioning of human BMSCs could influence their EV secretion. Sub-confluent primary hBMSCs cultured in FBS- or XFS-containing medium were maintained for 72 h in basal medium in an either normoxic or hypoxic environment. To exclude any negative effect on cell viability, early and late apoptotic cells were evaluated. In both normoxia and hypoxia, XFS-hBMSCs showed a higher percentage of live cells (89.9 ± 3.64% and 92.0 ± 4.61%, respectively) together with a decrease in early apoptotic cells (5.8 ± 0.40% and 4.94 ± 2.84), compared to FBS-hBMSCs (live cells: 67.16 ± 3.57% and 67.5 ± 7.99; early apoptotic cells: 26.8 ± 5.07% and 23.63 ± 4.30%) (p < 0.0001 for all the comparisons) (Figure 1A).

Collected conditioned media underwent differential ultra-centrifugations, to separate sEVs, obtaining normoxic and hypoxic FBS-hBMSC-derived sEVs (namely Normo FBS-sEVs and Hypo FBS-sEVs, respectively) and normoxic and hypoxic XFS-hBMSC-derived sEVs (namely Normo XFS-sEVs and Hypo XFS-sEVs, respectively). Protein content on the sEV surface was assessed by BCA assay. Notably, hypoxic preconditioning led to a significant enrichment of proteins on the hBMSC-sEVs surface in both FBS and XFS groups (1.14 ± 0.12 μg/10^6^ cells and 2.15 ± 0.61 μg/10^6^ cells, respectively) with an almost two-fold increase compared to normoxic condition (0.36 ± 0.16 μg/10^6^ cells and 1.15 ± 0.19 μg/10^6^ cells, p < 0.05 and p < 0.01, respectively, Figure 1B). In both normoxia and hypoxia, XFS-cultured hBMSCs showed an increase in sEV release compared to FBS (p < 0.05 and p < 0.01, respectively).

### Xeno-free culture enhanced EV secretion of hBMSCs

To detect particle concentration and size distribution, FBS- and XFS-sEVs both in normoxia and hypoxia were further analyzed via NTA using the Zetaview. No difference was observed in size distribution between the different experimental groups with particles showing an average size of 140-160 nm throughout (Figure 1C). The analysis also showed a reasonably high number of particles/cell in all the four conditions with an almost two-fold increase in XFS-sEVs, both in normoxia (303 ± 57.94 particle/cell) and hypoxia (328 ± 23.64 particle/cell) compared to FBS-sEVs (150 ± 3.77 particle/cell and 195 ± 19.03 particle/cell, respectively), Figure 1D. Contrary to BCA results, no significant difference was found in particle number between normoxic and hypoxic sEVs in both culture conditions (FBS and XFS). The ratio between the number of particles and protein content underlined that in both FBS- and XFS-sEVs, the hypoxic pre-conditioning did not induce a higher secretion of EVs, but rather a protein enrichment (Figure 1E).

**Figure 2:**
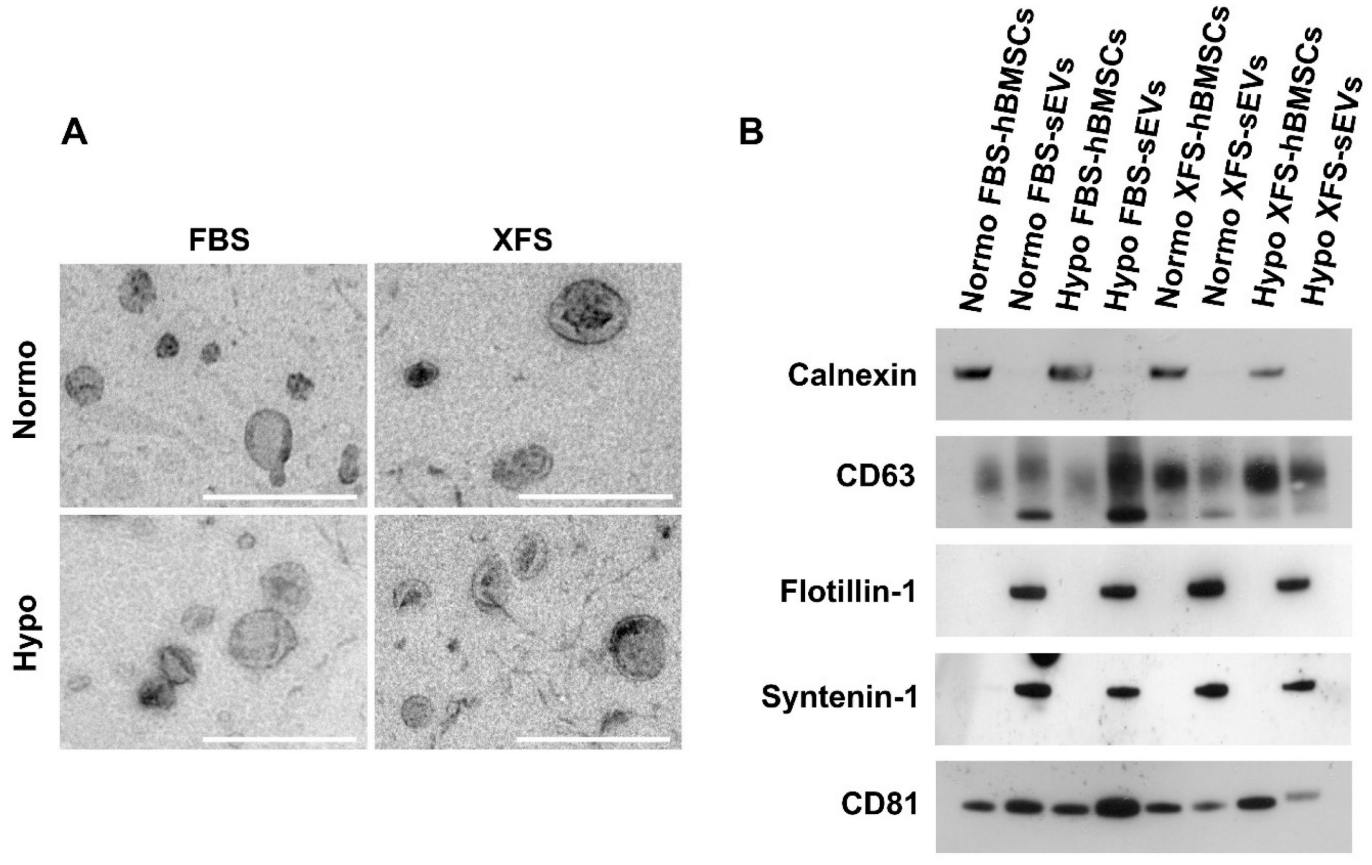
** Characterization of sEVs. (A)** TEM micrographs of isolated sEVs. Scale bar: 500 nm.** (B)** Western blot analysis on Normo and Hypo FBS- and XFS-sEVs. Control cell lysates were also loaded. Specific expressions of CD63, CD81, flotillin, syntenin and calnexin were investigated.

EVs were also investigated by TEM, to confirm the efficient isolation. They exhibited the typical morphology of vesicle surrounded by a lipidic bilayer (Figure 2A), with a low level of clustering in all the samples. EV characterization by Western blot revealed that both FBS- and XFS-sEVs expressed the specific EV markers, such as CD81 and CD63. However, Hypo FBS-sEVs showed a higher expression of both proteins compared to their normoxic counterpart, whereas in XFS-sEVs this increase was less prominent (Figure 2B). No CD9 expression was found in all the samples analyzed. Syntenin-1 and flotillin-1 were found to be abundant in both FBS-sEVs and XFS-sEVs. To rule out any contamination from other cellular compartments, the expression of the chaperon calnexin, an endoplasmic reticulum trans-membrane protein 20, was also analyzed. However, it was detected in cell lysate controls only (Figure 2B).

**Figure 3:**
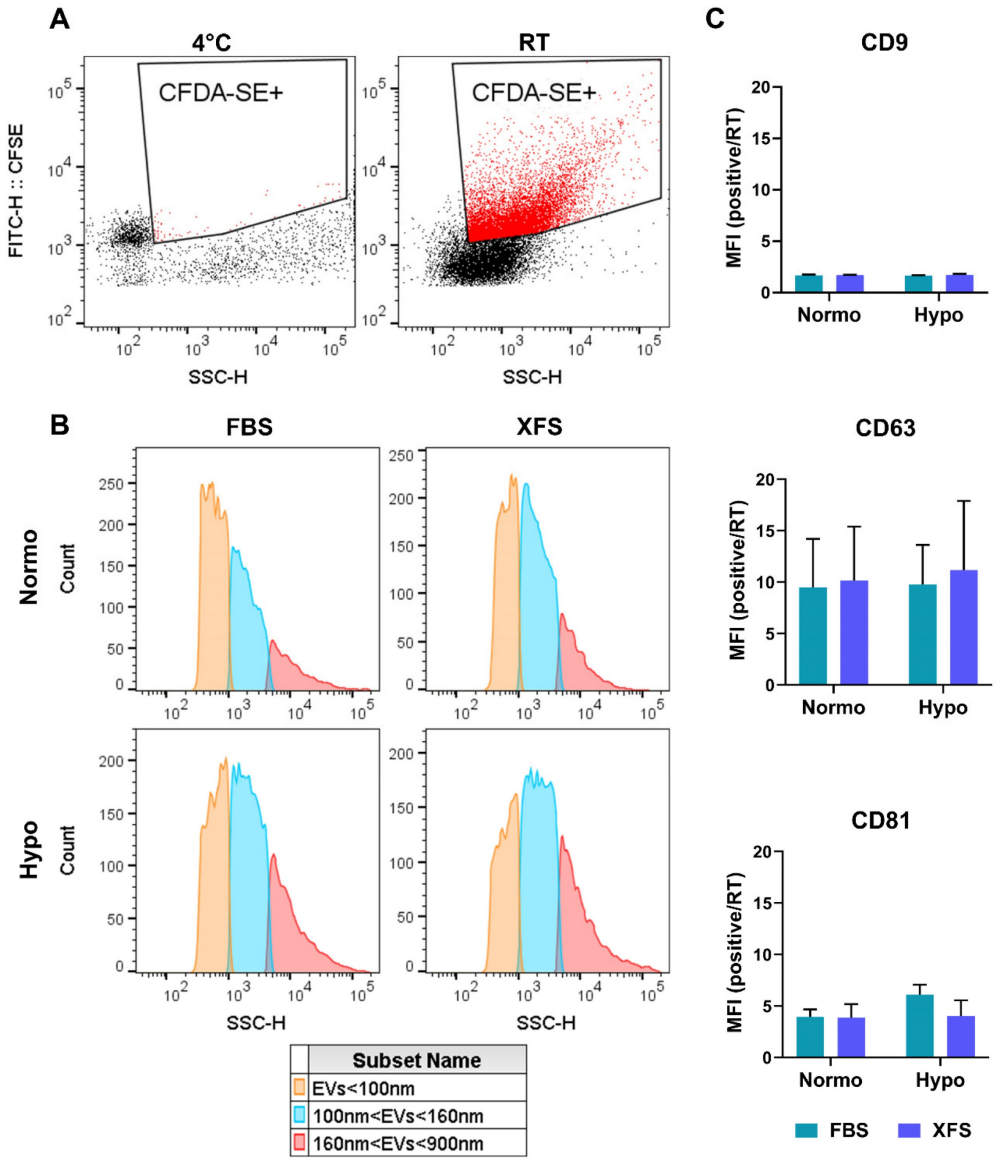
** Non-conventional flow cytometry strategy used to characterize sEVs. (A)** CFDA-SE staining for identification of intact vesicles. Red areas identify CFDA-SE positive events. EVs were stained with CFDA-SE at 4 °C as "blank tube" (left), useful to define the appropriate dimensional gate when considering EVs stained with CFDA-SE at room temperature (right). **(B)** Size distribution of EV subtypes. Three dimensional gates were considered: EVs ≤100 nm (orange), 100 nm ≤ EVs ≤ 160 nm (blue) and 160 nm ≤ EVs ≤ 900 nm (red). **(C)** Quantification of CD9-, CD63- and CD81- positive events falling within the CFDA-SE gate. Data are presented as ratio between mean fluorescence intensity (MFI) of sEVs stained with a specific antibody and MFI of correspondent isotype control (relative MFI). Data are representative of at least three independent experiments.

Subsequently, FBS- and XFS-sEVs were further characterized by non-conventional flow cytometry. To distinguish vesicles from background and debris, both FBS- and XFS-sEVs were stained with CFDA-SE fluorescent dye. Since, CFDA-SE diffuses within vesicles, where is cleaved by intra-vesicular esterase, becoming fluorescent only at room temperature (Figure 3A, right panel); a 4 °C control was also performed (Figure 3A, left panel). Considering the CFDA-SE positive events and using a mixture of 100-900 nm dimensional beads, three dimensional gates were identified as follows: i) EVs < 100 nm (small); ii) 100 nm < EVs < 160 nm (medium); iii) 160 nm < EVs < 900 nm (large). The percentage of CFDA-SE positive events in each gate was calculated (Figure 3B) (Table 2). Hypoxic sEVs showed a slight but not significant increase in medium (i.e., 52.90 ± 6.96% FBS; 53.56 ± 8.50% XFS) and large (i.e., 29.10 ± 4.22% FBS; 32.76 ± 12.83% XFS) EVs, compared to normoxic sEVs in both FBS (medium: 44.20 ± 7.13%; large: 17.93 ± 5.06%) and XFS-sEVs (medium: 48.10 ± 14.31%; large: 15.79 ± 8.22%). No difference was observed in the percentage of EVs < 100 nm between FBS- and XFS-sEVs, both in normoxia and hypoxia. The expression of CD9, CD63 and CD81 was also evaluated with Median Fluorescent Intensity (MFI) calculated and normalized to isotype controls (Figure 3C). According to western blot results, CD9 expression was very low in both Normo- and Hypo- FBS- and XFS-sEVs, while CD63 and CD81 expression was confirmed without any significant difference between the groups.

### XFS-sEVs are more efficiently internalized from OA-hACs

The development of OA is strictly related to the upregulation of pro-inflammatory cytokines 21 and MSC-derived EVs have recently been reported as cell-free anti-OA therapeutic strategy 22-25.

**Figure 4:**
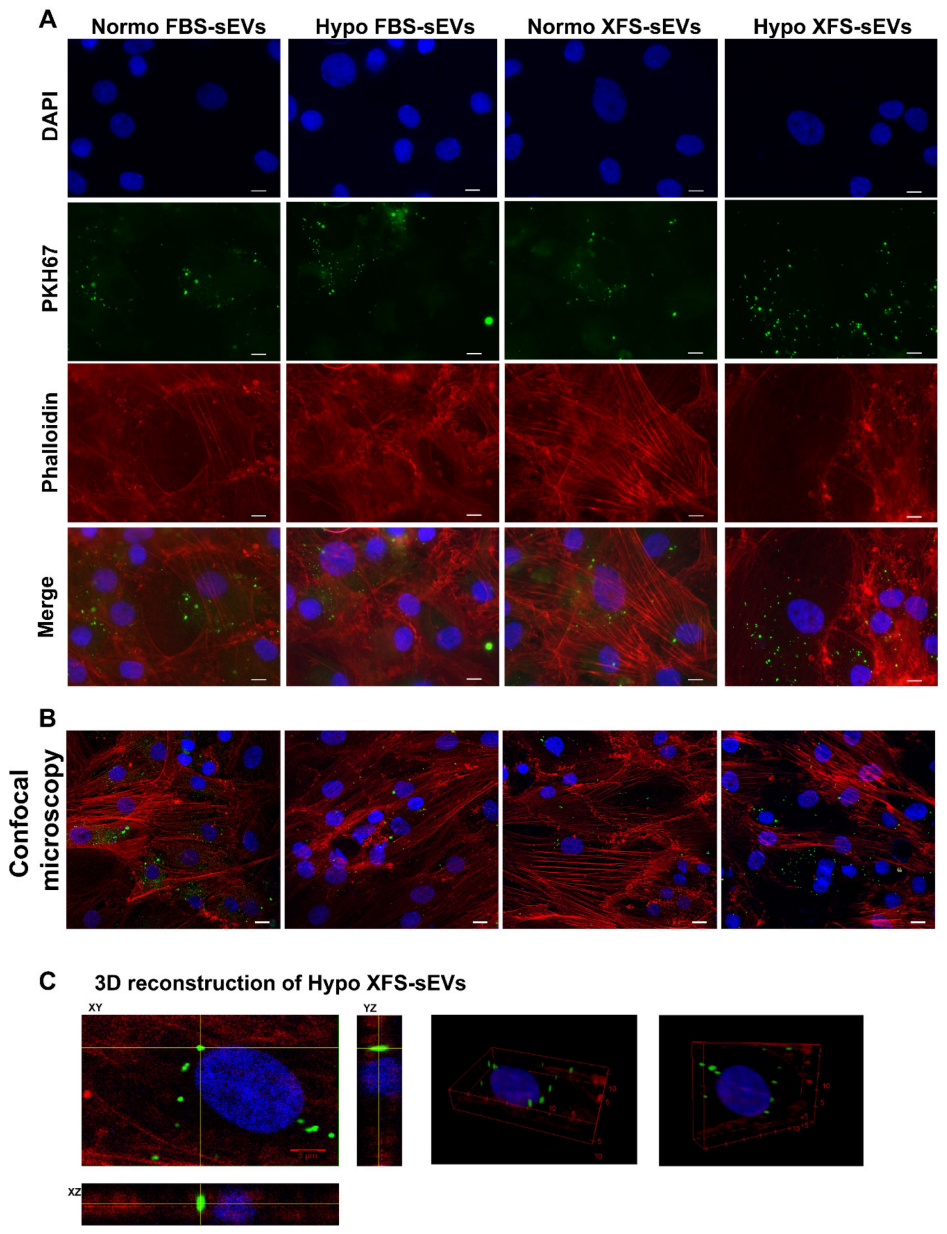
** Uptake of EVs on OA-hACs. (A)** Representative images of PKH67-stained sEVs internalized by OA-hACs. From the top, pictures show the nuclei (DAPI), sEVs (PKH67), actin cytoskeleton (Phalloidin) and merge. Scale bar: 20 μm. **(B)** Confocal microscopy. **(C)** 3D reconstruction.

To evaluate the role exerted by FBS- and XFS-sEVs in cartilage repair and OA, the interactions of sEVs with recipient cells was preliminarily investigated. Human articular chondrocytes (hACs) were treated with IL-1α to induce inflammation and mimic the OA environment. OA-hACs were subsequently incubated with PKH67-stained FBS- or XFS-sEVs derived from both normoxic and hypoxic hBMSCs. hACs efficiently internalized both FBS- and XFS-sEVs in normoxia and hypoxia within their cytoplasm (Figure 4A).

A further analysis by confocal microscopy was performed, to verify the effective uptake of sEVs and to exclude any event of sEV adhesion on the cell surface (Figure 4B). The three-dimensional reconstruction (Figure 4C) evidenced that hACs internalized EVs, localizing them around the nucleus, with a comparable average distance in all the conditions (Table 3). However, the calculated volumetric density (sEVs/mm^3^) showed that XFS-sEVs derived from hypoxic cells were internalized by hACs to a larger extent than FBS-hBMSC-EVs (Table 3).

### XFS-sEVs showed a strong anti-inflammatory effect

**Figure 5:**
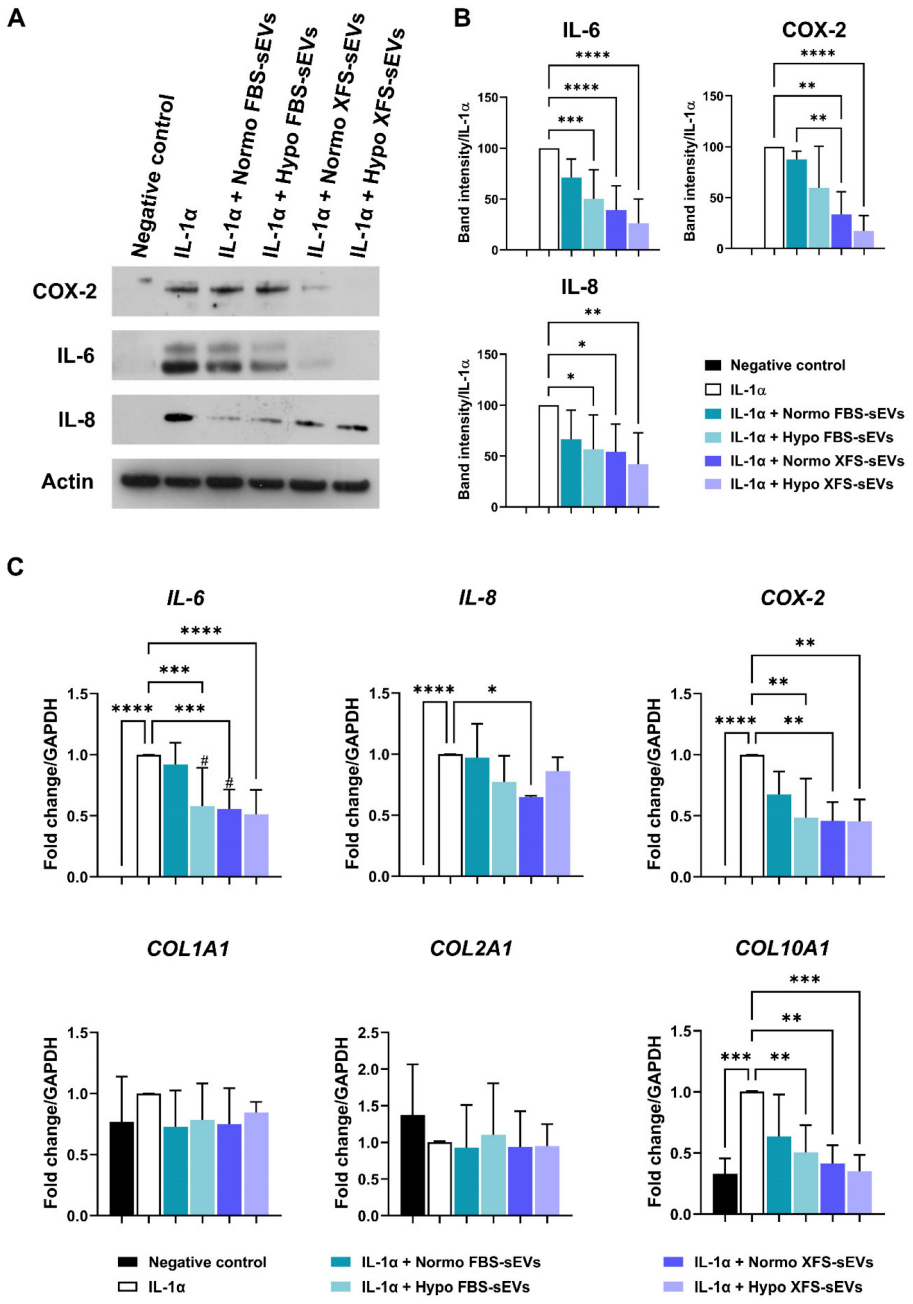
** Biological effect of sEVs on OA-hACs. (A)** Representative images of western blot of secreted IL-6 and IL-8 on conditioned media and COX-2 and actin of cell lysates of hACs treated with IL-1α ± Normo or Hypo FBS- or XFS-sEVs. **(B)** Quantification of band intensity of IL-6, IL-8 and COX-2. Intensity values were normalized to actin protein expression of the same experiment. Data are reported as normalized intensity on IL-1α positive control. Error bars represent S.D. (*) p < 0.05, (**) p < 0.01, (***) p < 0.001, (****) p < 0.0001, one-way ANOVA. Data are representative of four independent experiments. **(C)** Expression levels of *IL-6, IL-8, COX-2, COLL-I, COLL-II* and *COLL-X* quantified by real time-PCR of hACs treated with IL-1α ± Normo or Hypo FBS- or XFS-sEVs. Error bars represent S.D. (*) p < 0.05, (**) p < 0.01, (***) p < 0.001, (****) p < 0.0001, (#) p < 0.05 *vs.* Normo FBS-sEVs, one-way ANOVA. Data are representative of four independent experiments.

To investigate whether hBMSC-sEVs had an anti-inflammatory effect on OA-hACs, confluent hACs were treated for 16 h with IL-1α ± 1 μg/mL Normo or Hypo FBS-sEVs or XFS-sEVs. Modulation of IL-6, IL-8 and COX-2 was evaluated. As already reported 14, Normo FBS-sEVs exerted a blunt inhibitory effect on IL-6 and IL-8 secretion and COX-2 expression (Figure 5A). Hypoxia pre-conditioning allowed increased FBS-sEVs effectiveness, showing a significant decrease of IL-6 and IL-8 levels, compared to IL-1α treatment (p < 0.01 and p < 0.05, respectively). Additionally, a strong anti-inflammatory outcome was achieved by both Normo and Hypo XFS-sEVs, with a considerable weakening of IL-6 (p < 0.0001, in both Normo and Hypo) and IL-8 secretion (Normo: p < 0.05; Hypo: p < 0.01) and COX-2 expression (Normo: p < 0.01; Hypo: p < 0.0001) compared to IL-1α treatment. In addition, no significant differences were observed between Normo and Hypo XFS-sEVs treatments (Figure 5B).

Quantitative PCR on *IL-6, IL-8* and *COX-2* genes confirmed the strong anti-inflammatory effect of Hypo FBS-sEVs and Normo and Hypo XFS-sEVs, especially on weakening the *IL-6* (Hypo FBS sEVs and Normo XFS sEVs: p < 0.001; Hypo XFS sEVs: p < 0.0001) and *COX-2* expression (p < 0.01 in all the conditions, Figure 5C). Expression of collagens genes was also investigated (Figure 5C). Interestingly, *COL1A1* and *COL2A1* expressions were not significantly affected by IL-1α and sEVs treatment, compared to negative control. On the other hand, *COL10A1* showed a significant increase after IL-1α treatment, compared to untreated cells (p < 0.001). Normo FBS-sEVs exerted a weak not significant inhibitory effect on *COL10A1* expression, while hypoxia pre-conditioning increased FBS-sEVs effectiveness (p < 0.01), reflecting the biological effect observed on pro-inflammatory cytokines expression. Similarly, both Normo and Hypo XFS-sEVs showed a strong ability to revert *COL10A1* expression compared to IL-1α treatment (Normo: p < 0.01; Hypo: p < 0.001). Taken together, these results could suggest a beneficial role of sEVs in OA treatment.

### hBMSC-sEVs inhibited IL-1α-induced NF-κB activation and PGE2 production in OA-hACs

**Figure 6:**
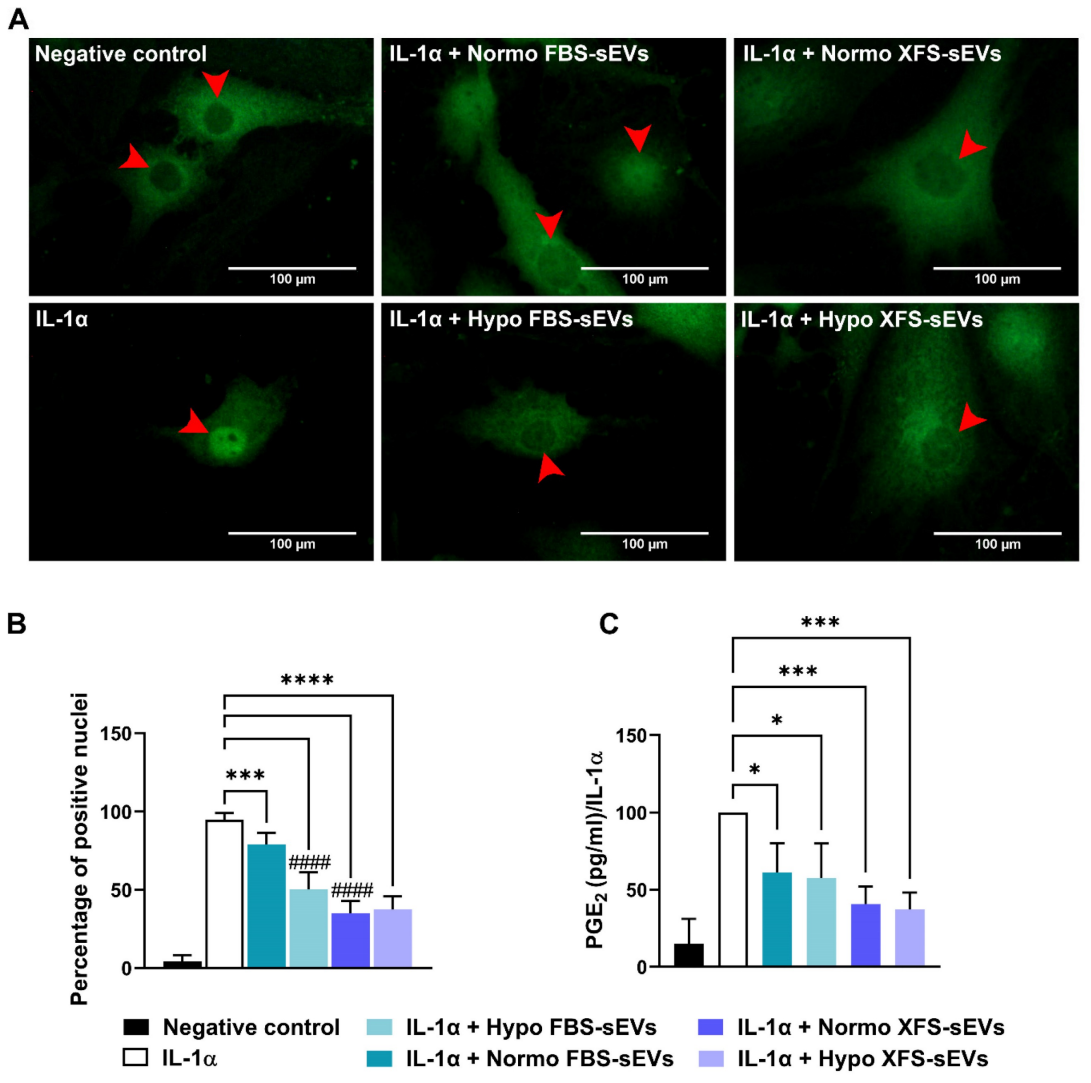
** Mechanism of action of sEVs on OA-hACs. (A)** NF-kB nuclear translocation in OA hACs in response to sEVs treatment. Red arrows show the nucleus of the cells. Images are representative of four independent experiments. Scale bar: 100μm. **(B)** Quantification of NF-kB nuclear translocation, calculated as number of cells with positive nuclei on total cell number. Error bars represent S.D. (***) p < 0.001, (****) p < 0.0001, comparison vs. IL-1a, (^####^) p < 0.0001, comparison vs. IL-1a + Normo FBS-sEVs, one-way ANOVA. **(C)** PGE_2_ ELISA quantification, expressed as percentage of positive control (IL-1α). Error bars represent S.D. (*) p < 0.05, (***) p < 0.001, one-way ANOVA.

NF-κB has been reported as a key player in the establishment of an inflammatory milieu 26,27. It induces the expression of pro-inflammatory cytokines and cyclooxygenase enzymes, such as COX-2, which convert arachidonic acid to PGE2, one of the main inflammatory mediators in OA 28. In this context, FBS- and XFS-sEVs were assessed for their ability to modulate NF-κB activity. Subcellular localization of NF-κB was evaluated by immunofluorescence, revealing that IL-1α treatment induced NF-κB nuclear translocation (Figure 6A). Even if no modulation was observed in IL-6, IL-8 and COX-2 expression, treatment with Normo FBS-sEVs partially reversed (~15%) IL-1α-induced NF-κB translocation (p < 0.001, Figure 6A-B). Comparable to western blot results, hypoxia pre-conditioning led to strengthen the inhibitory effect of FBS-sEVs, allowing a significant increase of NF-κB nuclear translocation (-45%, p < 0.0001 *vs.* IL-1α). A strong abolishment (~60%) was also achieved by both Normo and Hypo XFS-sEVs (p < 0.0001 *vs.* IL-1α). Similarly, to IL-6, IL-8 and COX-2 protein level reduction, no significant differences were observed between normoxic and hypoxic pre-conditioning in XFS-sEVs treatment.

PGE_2_ expression was also evaluated after treatment with IL-1α ± FBS- or XFS-sEVs (Figure 6C). Both Normo and Hypo FBS-sEVs were able to blunt PGE_2_ production (p < 0.05 *vs.* IL-1α), without significant differences between the two groups. Analogously to NF-κB modulation, XFS-sEVs both in normoxia and hypoxia exhibited a robust inhibitory effect on PGE_2_ secretion (p < 0.001 *vs.* IL-1α). No differences were observed between normoxia and hypoxia pre-conditioning.

These results suggest that both FBS- and XFS-sEVs could potentially affect OA-related inflammation.

### FBS- and XFS-sEVs exhibited a different miRNA cargo

**Figure 7:**
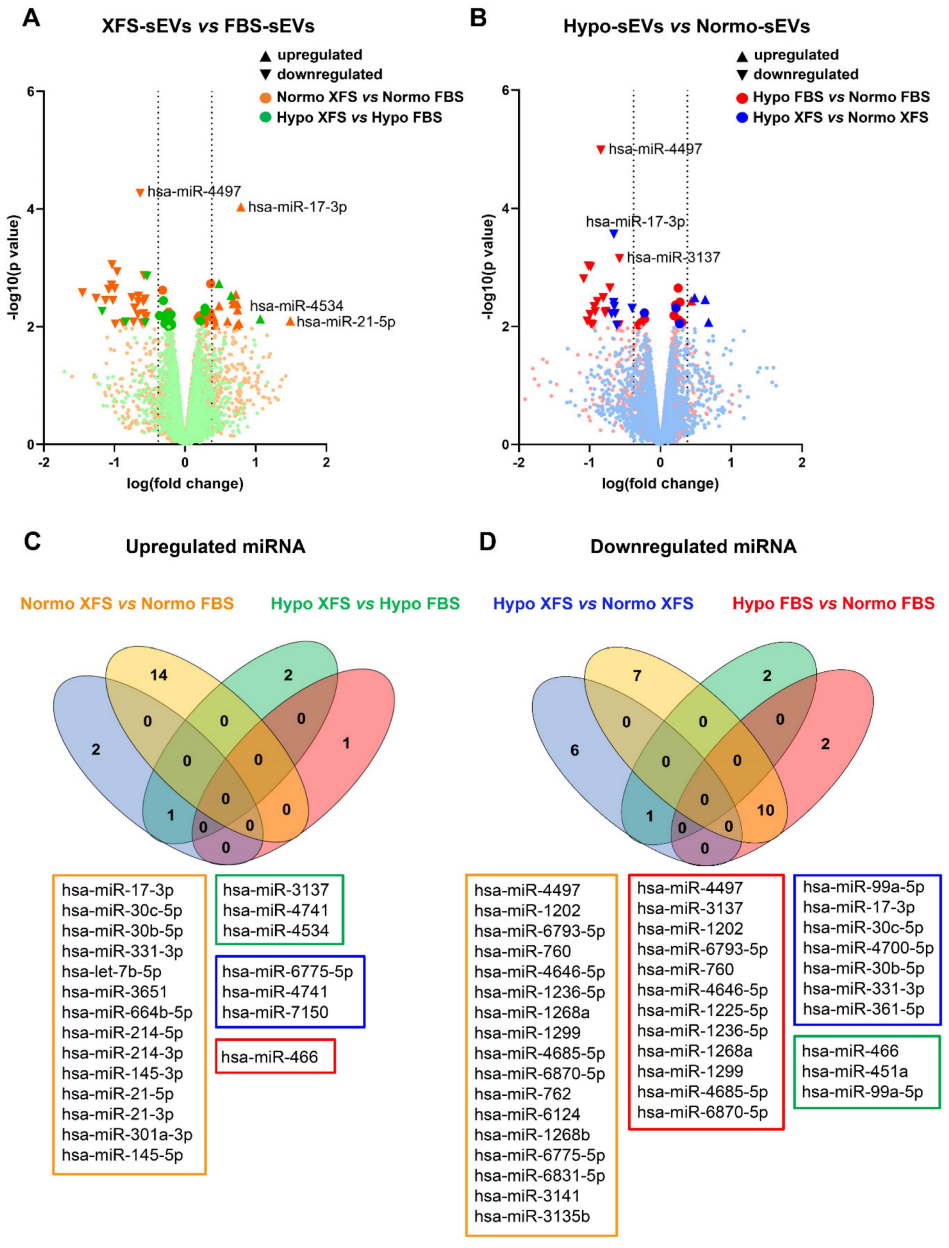
** Characterization of miRNA content of sEVs. (A)** Volcano plot showing differentially expressed miRNA in XFS- compared to FBS-sEVs, both in normoxia (orange) and hypoxia (green). **(B)** Volcano plot showing differentially expressed miRNA in Hypo- compared to Normo-sEVs, both in FBS (red) and XFS (blue). **(C)** Venn diagram showing upregulated miRNA in Normo XFS-sEVs *vs.* Normo FBS-sEVs (orange), Hypo XFS-sEVs *vs.* Hypo FBS-sEVs (green), Hypo XFS-sEVs *vs.* Normo XFS-sEVs (blue), Hypo FBS-sEVs *vs.* Normo FBS-sEVs (red). **(D)** Venn diagram showing downregulated miRNA in Normo XFS-sEVs *vs.* Normo FBS-sEVs (orange), Hypo XFS-sEVs *vs.* Hypo FBS-sEVs (green), Hypo XFS-sEVs *vs.* Normo XFS-sEVs (blue), Hypo FBS-sEVs *vs.* Normo FBS-sEVs (red).

EVs effect their biological function through the combined activity of various molecules, including miRNAs, which mediate EV effects on recipient cells regulating the expression of target genes 29. To investigate whether the preconditioning strategies had affected the miRNA cargo within both FBS and XFS-sEV, the expression profiles of 2,549 EV-miRNAs were evaluated by microarray. Specifically, we compared how Normo or Hypo pre-conditioning, as well as the culture supplements (*i.e.,* FBS and XFS), could influence sEV-miRNA enrichment (Figure 7).

The analysis revealed 14 upregulated (Figure 7A-C) and 17 downregulated (Figure 7A-D) miRNAs in XFS-sEVs compared to FBS-sEVS, in normoxia. Among the upregulated miRNAs, miR-17-3p and miR-21-5p were the most significantly overexpressed ones in XFS-sEVs in comparison with FBS-sEVs (Figure 7A-C).

Conversely, fewer EV-miRNAs were found differentially expressed in FBS and XFS-sEVs, after hypoxic preconditioning (Figure 7A, in green). Only three upregulated and three downregulated miRNAs were detected (Figure 7C-D). This data led to the hypothesis that hypoxic pre-conditioning lowered differences between XFS- and FBS-sEVs and may explain the dissimilar anti-inflammatory effect of Hypo and Normo FBS-sEVs described above.

Hypoxia and normoxia conditions were also compared for both FBS and XFS-sEVs (Figure 7B). Twelve miRNAs were less expressed in hypoxia compared to normoxia in FBS-sEVs (Figure 7D) and ten of these (*i.e.,* miR-4497, miR-1202, miR-6793-5p, miR-760, miR-4646-5p, miR-1236-5p, miR-1268a, miR-1299, miR-4685-5p, miR-6870-5p) were likewise downregulated in XFS-sEVs compared to FBS-sEVs in normoxia.

Finally, miRNA cargo of Hypo XFS-sEVs was also compared with Normo XFS ones and only three upregulated (*i.e.,* miR-6775-5p, miR-4741, miR-7150) and seven downregulated (*i.e.,* miR-99a-5p, miR-17-3p, miR-30c-5p, miR-4700-5p, miR-30b-5p, miR-331-3p, miR-361-5p) sEV-miRNAs were observed (Figure 7C-D).

### XFS-sEVs selected a miRNA cargo involved in cartilage homeostasis

**Figure 8:**
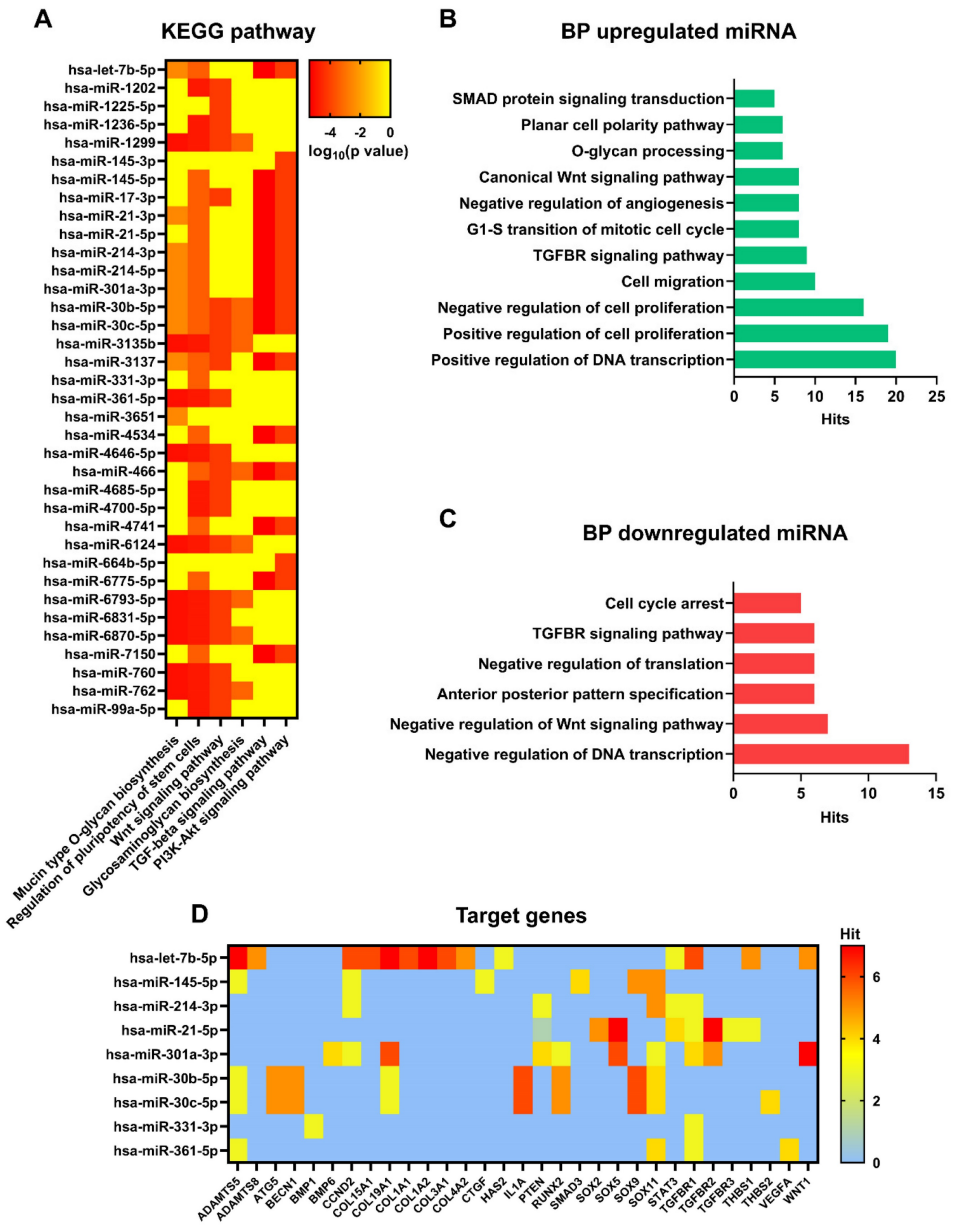
** Pathway, biological processes, and target genes of expressed miRNA. (A)** Heat map showing main pathways regulated by expressed miRNA, analysed with DIANA myRPath 3.0. Colour scale is representative of log_10_ (p value). **(B)** Biological processes of upregulated miRNA, analysed with DIANA myRPath 3.0. **(C)** Biological processes of downregulated miRNA, analysed with DIANA myRPath 3.0. **(D)** Heat map showing target genes regulated by expressed miRNA, analysed with miRsystem. Colour scale is representative of hits number.

To better understand the role of differentially expressed miRNAs in sEV biological activity and their role in anti-inflammatory activity, a gene ontology analysis based on *in silico* predicted target genes, was carried out (Figure 8A). Eight of the ten miRNAs downregulated in Normo XFS-sEVs and Hypo FBS-sEVs compared to Normo FBS-sEVs (miR-1202, miR-1236-5p, miR-1299, miR-3137, miR-4646-5p, miR-6793-5p, miR-6870-5p, miR-760) were commonly involved in mucin type O-glycan biosynthesis, regulation of pluripotency of stem cells and the Wnt signaling pathway.

On the other hand, miR-21, miR-145, miR-214, miR-301a-5p and let-7b-5p, which were upregulated in XFS-sEVs compared to FBS-sEVs in normoxia, were predominantly implicated in TGF-beta and PI3K-Akt signaling pathways. Both miR-30c-5p and miR-30b-5p were found to be involved in all pathways mentioned above and in glycosaminoglycan biosynthesis to a limited extent.

In addition, gene ontology also revealed that upregulated miRNAs were generally involved in positive regulation of DNA transcription, cell proliferation, cell migration, cell cycle, as well as TGF-beta receptor and Wnt signaling pathways, as well as the cell polarity (Figure 8B). Contrarily, downregulated miRNAs were mostly related to negative regulation of DNA transcription and translation, negative regulation of Wnt signaling pathway and cell cycle arrest (Figure 8C).

Among the genes targeted by upregulated miRNAs in XFS-sEVs (let-7b-5p, miR-145-5p, miR-30b and miR-30c), genes related to cartilage homeostasis emerged, such as *ADAMTS5* and *ADAMTS8* (A Disintegrin and Metalloproteinase with Thrombospondin motifs 5 and 8, respectively) (Figure 8D). Notably, these genes are also well known to be involved in OA pathogenesis and to facilitate aggrecan degradation 30. In addition, miR-21-5p, which was one of the most upregulated miRNAs in XFS-sEVs, showed associations with *SOX* genes (SRY-Box Transcription Factor), together with miR-145-5p, miR-214-3p, miR-301a-3p, miR-30b and miR-30c. *SOX* genes are master transcription factors in chondrocytes, with an essential role in cartilage development and lineage specification 31. Let7b-5p also targets some collagen genes, such as *COL15A1* (type 15 collagen, alpha 1), *COL19A1* (type 19 collagen, alpha 1), *COL1A1* (type 1 collagen, alpha 1), *COL1A2* (type 1 collagen, alpha 2), *COL3A1* (type 3 collagen, alpha 1), *COL4A1* (type 4 collagen, alpha 1). Further target genes which might be related to cartilage homeostasis were *HAS2* (Hyaluronan Synthase 2, target of let-7b-5p), which is the main enzyme involved in hyaluronic acid production 32 and *THBS1* (Thrombospondin 1, target of let-7b-5p and miR-21-5p), well known for its anti-angiogenic and anti-inflammatory effects 33. *TGFBR* (Transforming Growth Factor Beta Receptor) was also identified as target gene for several EV-miRNAs (let-7b-5p, miR-214-3p, miR-21-5p, miR-301a-3p, miR-331-3p, miR-361-5p), together with the Wnt/ β-catenin signaling pathway effector *WNT1* (Wnt Family Member 1). Additionally, miR-145-5p showed interactions also with CTGF (Connective Tissue Growth Factor), a key molecule often associated with connective tissues regeneration and wound healing 34.

Additional target genes found from the analysis were *BMP1* (Bone Morphogenetic Protein 1, target for miR-331-3p), *BMP6* (Bone Morphogenetic Protein 6, target for miR-301a-3p) and *RUNX2* (Runt-related transcription factor 2, target for miR-301a-3p, miR-30b-5p and miR-30c-5p). In particular, *BMPs* are multi-functional growth factors with roles in embryonic development 35, while *RUNX2* is a transcription factor essential for osteoblast differentiation and chondrocytes maturation during endochondral ossification 36. Moreover, miR-30b-5p and miR-30c-5p can also target *ATG5* (Autophagy-Related Gene 5) and *BECN1* (Beclin 1), which are master genes of autophagy 37, and *IL-1α*. All together these data suggest that XFS-sEVs segregate a miRNA cargo exploiting a more chondroprotective and anti-inflammatory effect.

### MiR-145 and miR-214 showed anti-inflammatory properties

**Figure 9:**
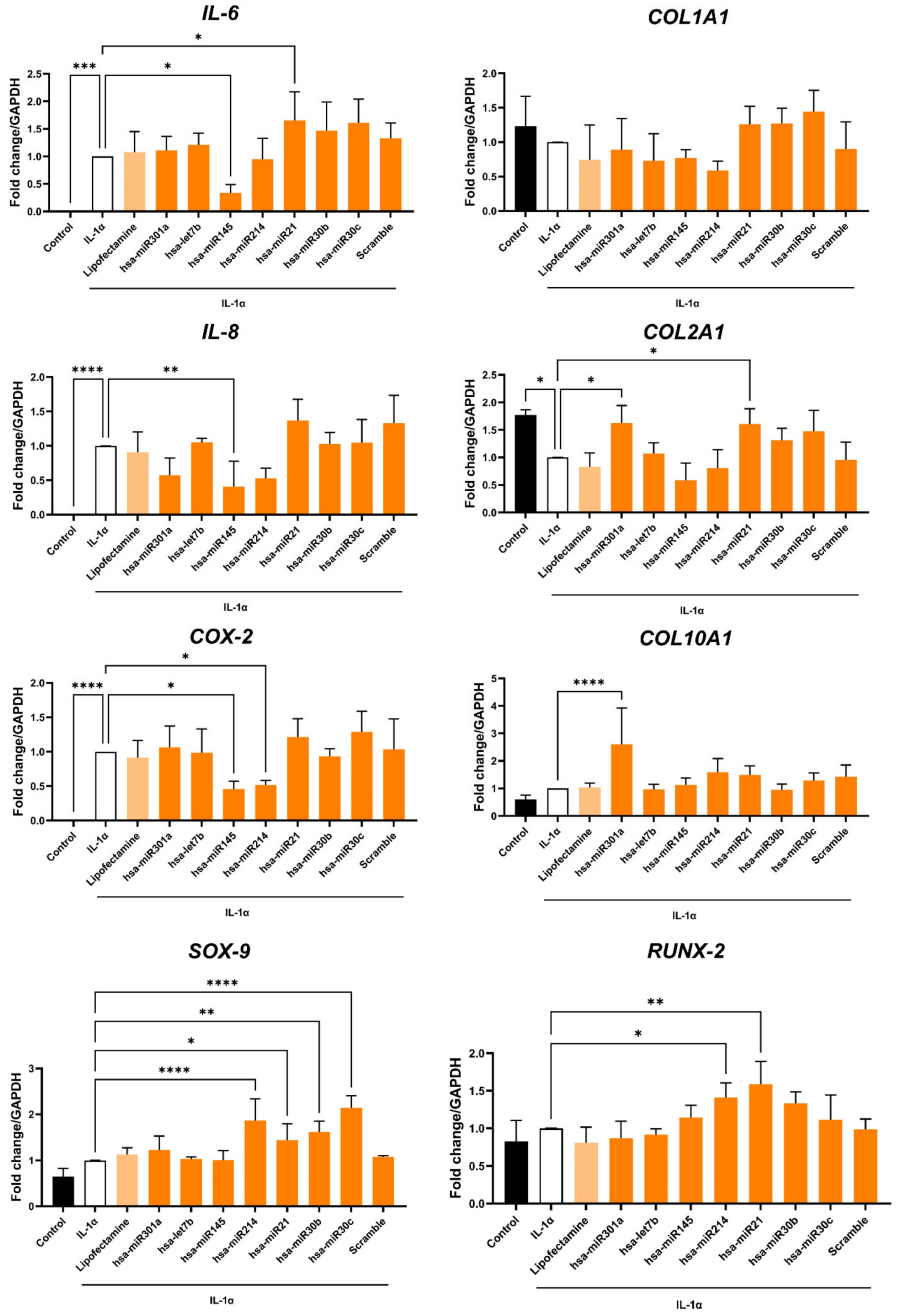
** Functional study of selected miRNA in OA-hACs**. Human articular chondrocytes were transfected with 100nM hsa-miR301a, hsa-let-7b, hsa-miR145, hsa-miR214, hsa-miR21, hsa-miR30b, hsa-miR30c or with a scramble control. Graphs show the relative expression of target genes *IL-6, IL-8, COX-2, COLL-I, COLL-II, COLL-X, SOX9* and* RUNX-2.* Error bars represent S.D. (*) p < 0.05, (**) p < 0.01, (****) p < 0.0001, one-way ANOVA. Data are representative of five independent experiments.

Based on gene ontology analysis, EV-encapsulated miRNAs targeting cartilage related genes (i.e., let-7b-5p, miR-145-5p, miR-214-3p, miR-21-5p, miR-301a-3p, miR-30b-5p and miR-30c-5p) were functionally validated in an OA *in vitro* environment (Figure 9). HACs were transfected with the selected miRNA mimics and then inflammation was triggered by IL-1α treatment. Interestingly, miR-145 emerged as a strong anti-inflammatory effector. Its overexpression led indeed to a significant down-regulation of *IL-6* (p < 0.05), *IL-8* (p < 0.01) and *COX-2* (p < 0.05) expression (Figure 9, left panel). In addition, miR-214 was also found to induce a slight not significant inhibition of *IL-8* expression and an effective reversion of *COX-2* gene levels (p < 0.05) (Figure 9, left panel).

On the other hand, miR-21 showed an opposite behavior, determining an increase of pro-inflammatory cytokines expression, in particular *IL-6* (p < 0.05). However, miR-21 was also able to selectively induce an upregulation of the chondrocytes master gene *SOX9* (p < 0.05) and the transcription factor *RUNX2* (p < 0.01). In addition, *SOX9* was also strongly upregulated after transfection with miR-214 (p < 0.0001), miR-30b (p < 0.01) and miR-30c (p < 0.0001), according to gene ontology analysis (Figure 8D). Furthermore, miR-214 overexpression induced *RUNX2* gene expression (p < 0.05).

Finally, the expression levels of collagen genes were also investigated, showing significant deregulations after miRNA transfections. Interestingly, *COL2A1* (type II collagen), a specific marker in both hyalin and articular cartilage, was significantly downregulated under the inflammatory stimulus compared to non-transfected negative control (p < 0.05). On the contrary, *COL1A1* (type I collagen) did not show significant variations after transfections with selected miRNA. Transfection with miR-301a or miR-21 was able to protect chondrocytes by IL-1α-induced *COL2A1* expression (p < 0.05 in both conditions). On the other hand, miR-301a also determined a sustained increase in *COL10A1* (type X collagen, p < 0.0001), a typical marker of hypertrophic chondrocytes.

Taken all together, these results suggested that XFS- sEVs anti-inflammatory properties are not attributable to a single miRNA, but rather to an *orchestra* of EV-encapsulated miRNAs exerting a synergic effect, by targeting different pathways.

## Discussion

Recent studies have shown the effectiveness of MSCs in cartilage repair and OA-related symptom relief 1. However, limited evidence demonstrated that injected MSCs directly contribute to joint tissue restoration. Notwithstanding, they could influence the surrounding environment and mediate endogenous tissue repair by paracrine activity, fulfilled through the secretion of soluble and EV-encapsulated bioactive factors 38-40.

EVs are small, membrane-enclosed particles released by nearly all type of cells and generated either from cell membrane budding (microvesicles) or endosomal compartments (exosomes) 41. They incorporate biologically active signaling molecules, acting as innovate players in intercellular communication 42. The cargo and function of EVs are recurrently influenced by the pathophysiological state of secreting cells and the microenvironment 41. In this scenario, an appropriate preconditioning may prompt cells to execute the most suitable therapeutic response. Several methods, as low oxygen tension, starvation or inflammatory stimuli have been reported as strategies to mimic the typical environment of injury sites 12. Hypoxic conditioning has been proven to enhance proliferation of MSCs without loss of multi-lineage differentiation capabilities 18. It has also been reported that hypoxia can impacts on EV secretion in adipose tissue-derived MSCs 43, as well as in breast cancer cells like MCF-7 44 and in endothelial cells 45.

This study focused on paracrine functionality of hBMSCs cultured in a xeno-free culture system, in pursuance of a clinical application. This would allow to obtain a clean, safe and ready-to-use clinical grade product. EVs were separated and characterized and as previously described 14, XFS allows the isolation of cells secreting a higher amount of EVs, although the typical vesicular marker did not significantly vary compared to the control culture condition. Here hypoxic pre-conditioning did not affect the number of EVs secreted, but rather the protein amount, in line with previous data reported for amniotic fluid stem cells derived EVs 46.

Hypoxia was also recently reported to enhance the cartilage repair effect of MSC-EVs 47,48. According to these recent findings, the anti-inflammatory effect of FBS- and XFS-sEVs was evaluated in an *in vitro* model, using IL-1α to simulate the inflammation occurring during OA. Hypo FBS-sEVs showed a superior inhibitory effect on the production of pro-inflammatory cytokines in OA-chondrocytes, compared to Normo FBS-sEVs, confirming previous evidence.

On the other hand, the XFS culture condition selected MSCs secreting EVs with strong anti-inflammatory properties, which are not enhanced by hypoxic pre-conditioning. The anti-inflammatory effect is also supported by the observation that XFS-sEVs are more efficiently internalized from OA-hACs. This different behavior could be related to a distinctive cargo selection. MiRNA content of hypoxic FBS-sEVs showed indeed a similar profile to XFS-sEVs, both exhibiting a downregulation of miRNAs involved in mucin type-O glycan biosynthesis. It has been indeed reported that the glycosylation signature of chondrocytes and extracellular matrix composition are altered during OA progression 49. In healthy cartilage, a complicated balance exists between new matrix deposition and its digestion. In OA establishment, articular cartilage degradation includes an anabolic phase initially where chondrocytes struggle to repair the damage, depositing an impaired extracellular matrix. A subsequent catabolic phase by matrix digestion leads to cartilage erosion 50.

Furthermore, miRNAs involved in Wnt, TGF-beta and PI3K-Akt signaling pathways were upregulated in XFS-sEVs. More specifically, miR-21, let-7b-5p, miR-301a-3p, miR-214, miR-145, miR-30b-5p and miR-30c-5p expression was higher in XFS-sEVs miRNome compared to FBS-sEVs and their specific function was validated in chondrocytes target cells. Interestingly, chondrocytes transfection with miR-145 or miR-214 mimic protected chondrocytes from IL-1α induced pro-inflammatory cytokines expression (*IL-6, IL-8, COX-2*). MiR‑145 has been shown to regulate chondrocyte homeostasis, although its role in OA remains to be elucidated. MiR-145 was found downregulated in OA samples and IL-1β-induced chondrocytes and its upregulation improved chondrocyte viability, inhibiting cartilage ECM degradation 51. This study shows for the first time a direct anti-inflammatory effect of miR-145. These data support the involvement of EV-encapsulated miR-145-5p in cartilage homeostasis and its potential role in OA therapy.

According to our results on chondrocytes, miR-214 has been reported to inhibit inflammatory factors (TNF‑α, IL‑1β, IL‑6 and IL‑18) in TNF‑α‑treated vascular endothelial cells through down-regulation of PTEN and NF‑κB protein expression 52. Among miR-214 target genes, the skeletal key players *SOX9* and *RUNX2* were particularly relevant. *SOX9* is unmistakably recognized as a master transcriptional factor of cartilage differentiation and development 31, while *RUNX2* is essential for osteoblast differentiation and chondrocyte maturation. Different studies have indeed confirmed that an abnormal expression of miR-214 could determine bone and cartilage development disorders and lead to the onset of OA 53. These data suggest a dual role of miR-214 both in OA prevention and chondrogenesis.

Additionally, our results also demonstrated that transfection of chondrocytes with miR-21 mimic induced *SOX9* and *RUNX2* expression and preserved cells by IL-1α-induced *COL2A1* downregulation, suggesting a protective role in OA pathogenesis. It has been indeed already reported that up-regulation of miR-21-5p in IL-1β-treated chondrocytes increased *COL2A1* expression and decreased *MMP13* and *ADAMTS5* levels 54. Nevertheless, miR-21 overexpression also led to an enhanced expression of pro-inflammatory cytokines, such as *IL6*. Further studies are needed to better elucidate the role of miR-21 in OA.

Expression of *COL2A1,* together with *COL10A1*, was also upregulated by miR-301a. Type II collagen is massively synthesised by resting chondrocytes in the growth plate, whereas type X collagen is produced by hypertrophic chondrocytes before mineralisation of the ECM occurs 55. Alterations in expression of *COL2A1* or *COL10A1* could be related to impairment and degradation of hyalin cartilage or altered subchondral bone formation, respectively. These results could indicate a putative role of miR-301a in joint homeostasis and maintenance.

Interestingly, let-7b-5p did not show modulations on analysed genes. However, these analyses unveil some target genes of let-7b-5p, such as *ADAMTS5*, *ADAMTS8*, *TGFBR1*, *WNT1* and some fibrotic collagens (e.g. *COL19A1*, *COL15A1*). In detail, *ADAMTS5* is unequivocally implicated in OA pathogenesis through degradative activity on aggrecan, one of the major structural components of articular cartilage. Several ADAMTS proteases cleave the aggrecan core protein, compromising joint mechanical properties and exposing other structural molecules, such as type II collagen, to proteolysis 56.

Finally, miR-30b-5p and miR-30c-5p emerged for their target genes profiles. Both were shown to target *SOX9*, inducing its upregulation. Interestingly, miR-30b and miR-30c also target *ATG5* and *BECN1*, master regulators of autophagy. Autophagy is a cellular homeostasis mechanism, supporting cell survival under stress-induced conditions such as nutrient deprivation, hypoxia, and injury. This catabolic process regulates energy and nutrients through the removal of damaged or dysfunctional proteins and organelles. In articular cartilage tissue, the resident chondrocyte population cannot be replenished via vasculature. Autophagy is thus essential for maintaining cell and tissue homeostasis 57,58.

Taken together, presented data indicated that XFS culture medium is a relevant tool to obtain clean and ready-to-use cells and cellular products, amenable for clinical treatment of diseases such as OA, thanks to their miRNAs content. This study showed for the first time a potential role of EV-encapsulated miR-145 and miR-214 in blunting OA-related inflammation. However, the absence of an in vivo validation represents a limitation for the study and further experiments are ongoing to fill the research gap and confirm the presented data in an osteoarthritic mouse model.

## Figures and Tables

**Table 1:** Primers used for Evaluating the gene expression of human articular chondrocytes by quantitative Real-Time Polymerase Chain Reaction.

	Forward 5'	Reverse 5'
***IL-6***	TGACGACCTAAGCTGCACTT	GGGCTGATTGGAAACCTTATTA
***IL-8***	CGTGGCTCTCTTGGCAGC	TTAGCACTCCTTGGCAAAACTG
***COX-2***	AATTGCTGGCAGGGTTGCT	GGTCAATGGAAGCCTGTGATACT
***COL1A1***	CAGCCGCTTCACCTACAGC	TTTTGTATTCAATCACTGTCTTGCC
***COL2A1***	GGCAATAGCAGGTTCACGTACA	CGATAACAGTCTTGCCCCACTT
***COL10A1***	TGGGTAGGCCTGTATAAGAACGG	CATGGGAGCCACTAGGAATCCTGAGA
***RUNX2***	CGCAAAACCACAGAACCACAAGTGCG	GTTGGTCTCGGCTGGTAG
***SOX9***	CCCGCACTTGCACAACG	TCCACGAAGGGCCGCT
***GAPDH***	CCATCTTCCAGGAGCGAGAT	CTGCTTCACCACCTTCTTGAT

**Table 2:** Percentage of CFDA-SE positive events in the three considered size gates (Small: EVs < 100 nm; Medium: 100 nm < EVs < 160 nm; Large: 160 nm < EVs < 900 nm) calculated for both FBS- and XFS-sEVs in normoxia and hypoxia.

	Normo FBS-sEVs	Hypo FBS-sEVs	Normo XFS-sEVs	Hypo XFS-sEVs
***Small***	35.32 ± 9.90%	30.26 ± 11.56%	36.50 ± 24.67%	21.22 ± 15.41%
***Medium***	44.20 ± 7.13%	52.90 ± 6.96%	48.10 ± 14.31%	53.56 ± 8.50%
***Large***	17.93 ± 5.06%	29.10 ± 4.22%	15.79 ± 8.22%	32.76 ± 12.83%

**Table 3:** Quantitative analysis based on confocal microscopy.

	Average distance (μm)	Volumetric density (n° EVs/mm^3^)
**Normo FBS-sEVs**	12.7 ± 3.3	(7.0 ± 1.4) x 10⁵
**Hypo FBS-sEVs**	13.1 ± 2.6	(7.5 ± 1.2) x 10⁵
**Normo XFS-sEVs**	12.5 ± 2.6	(8.0 ± 1.4) x 10⁵
**Hypo XFS-sEVs**	12.7 ± 1.9	(13.5 ± 2.8) x 10⁵

## Abbreviations

BCA: bicinchoninic acid
CFDA-SE: carboxyfluorescein diacetate succinimidyl ester
CM: conditioned medium
COL1A1: Type I Collagen
COL2A1: Type II Collagen
COL10A1: Type X Collagen
COX-2: Cyclooxygenase 2
EDTA: ethylenediaminetetraacetic acid
EVs: extracellular vesicles
FBS: foetal bovine serum
hACs: human articular chondrocytes
hBMSCs: human bone marrow stromal cells
IL-1α: interleukin 1 alpha
IL-6: interleukin 6
IL-8: interleukin 8
MSC: mesenchymal stromal cell
NTA: nanoparticle tracking analysis
OA: osteoarthritis
PBS: phosphate buffer saline
PGE2: prostaglandin E2
SD: standard deviation
SOX9: SRY-box transcription factor 9
RUNX2: Runt-related transcription factor 2
TEM: transmission electron microscopy
XFS: xeno-free supplement
